# Effect of Three Training Systems on Grapes in a Wet Region of China: Yield, Incidence of Disease and Anthocyanin Compositions of *Vitis vinifera* cv. Cabernet Sauvignon

**DOI:** 10.3390/molecules201018967

**Published:** 2015-10-19

**Authors:** Mei-Ying Liu, Ming Chi, Yong-Hong Tang, Chang-Zheng Song, Zhu-Mei Xi, Zhen-Wen Zhang

**Affiliations:** 1 College of Enology, Northwest A & F University, Yangling 712100, Shannxi, China; E-Mails: qq493235763@163.com (M.-Y.L.); chiming101@163.com (M.C.); tangyonghong255@sina.com (Y.-H.T.); scz1103@gmail.com (C.-Z.S.); 2 Shaanxi Engineering Research Center for Viti-Viniculture, Yangling 712100, Shannxi, China

**Keywords:** training systems, grapes, yield, disease incidence, anthocyanin compositions

## Abstract

Grapevine training systems determine the suitability for grape varieties in a specific growing region. We evaluated the influence of three training systems, Single Guyot (SG), Spur-pruned Vertical Shoot-Positioned (VSP), and Four-Arm Kniffin (4AK), on the performance of grapes and vines of *Vitis vinifera* L. cv. Cabernet Sauvignon in the 2012 and 2013 growing seasons in a wet region of central China. 4AK was the most productive system in comparison to SG and VSP. SG and VSP had lower disease infections of leaves and berries, especially in the mid- and final stage of berry ripening. Three training systems had no impact on berry maturity. PLS-DA (Partial Least Squares-Discriminant) analysis showed that the relatively dry vintage could well discriminate three training systems, but the wet vintage was not. A wet vintage of 2013 had more accumulation of 3′5′-substituted and acylated anthocyanins, including malvidin-3-*O*-(6-*O*-acetyl)-glucoside, malvidin-3-*O*-glucoside, and petunidin-3-*O*-(*cis*-6-*O*-coumaryl)-glucoside, *etc.* With regard to the effect of training systems, 4AK grapes had the lowest concentrations of total anthocyanins and individual anthocyanins, SG and VSP differed according to the different vintages, and showed highest concentration of total individual anthocyanins in 2012 and 2013, respectively. Generally, VSP benefited the most, contributing to significantly highest levels of total individual anthocyanins, and major anthocyanin, including malvidin-3-*O*-glucoside and malvidin-3-*O*-(6-*O*-acetyl)-glucoside, and the grapes obtained from VSP presented significantly highest proportion of 3′5′-substituted anthocyanins. With regard to the ratios of 3′5′/3′-substituted, methoxylated/non-methoxylated and acylated/non-acylated anthocyanins, the significantly higher levels were also shown in VSP system. In summary, VSP was the best training system for Cabernet Sauvignon to accumulate relatively stable individual anthocyanins in this wet region of China and potentially in other rainy regions.

## 1. Introduction

The climate of a region of grape cultivation is widely recognized as a primary driver of grape quality. Climate change has a strong impact on weather patterns of grape regions, which will determine whether there are suitable for specific types of wines or not [1]. The grape and wine industry in China has continually developed in recent years, mostly on the west coast near the Pacific Ocean, which enjoys a marked continental monsoonal climate. The climate in these areas is humid and rainy throughout berry development, which is unfavourable for optimal grape growth, sugar accumulation, degradation of organic acids, and formation of phenolic compound necessary for the production of quality wines [2]. The incidence of disease in these areas is also an extremely serious problem during the berry-growing season.

Horticultural practices, such as canopy management, can be optimised to adjust berry and wine quality for helping to overcome the disadvantages of the local climatic conditions [1]. Training systems, which can strongly influence microclimatic conditions, have received attention because they directly impact temperature, humidity, and other environmental factors [3,4]. The diversity of grapevine training systems has arisen from differences between the growth habits and cropping capacities of grape species and varieties and from environmental and economic constraints on vineyard management [5]. Training systems should ideally be labour-efficient and adapted to the local climate [6]. Choosing the most efficient training system for the production of fruit of a desired quality in an unfavourable climatic region is thus vital.

Anthocyanins, a class of phenolic compounds important in wine production, contribute most of the orange, pink, red, blue, and purple colours to grapes and their wines [7,8,9]. Anthocyanins are important parameters of wine quality and directly influence the organoleptic characteristics of wines, such as colour and astringency [10]. They accumulate after véraison via the phenylpropanoid biosynthetic pathway in the grape skin [11], where they serve a wide range of biological functions such as protection against solar exposure and ultraviolet radiation, free-radical scavenging and anti-oxidative capacity, defence against a variety of pathogens, attraction of predators for seed dispersal, and the newly proposed modulation of signalling cascades [12,13,14]. The accumulation and metabolism of anthocyanins in grape skins are complex physiological and biochemical processes [15], and anthocyanin content depend on climatic and geographical factors, cultural practices, and grape cultivars [16,17,18,19,20]. Vine training systems can influence vine performance, such as vine size, canopy density, grape maturity, and berry quality [21,22,23,24]. The influence of training systems in unfavourable climatic regions, such as wet areas, on grape diseases and anthocyanin profiles in the skins of Cabernet Sauvignon grapes (*Vitis vinifera* L.), however, has not been well studied.

Single Guyot (SG), Spur-pruned Vertical Shoot-Positioned (VSP), and Four-Arm Kniffin (4AK) in this study have been the traditional training systems applied in China, the objective of this study was to evaluate the influence of three training systems on the yield and disease incidence of vines and on the anthocyanin composition of Cabernet Sauvignon grapes in a wet region of Shaanxi Province in central China. This research could have practical applications in vineyard management and will provide information for the production of high-quality wine in this and other rainy regions.

## 2. Results and Discussion

### 2.1. Climatic Conditions

**Figure 1:**
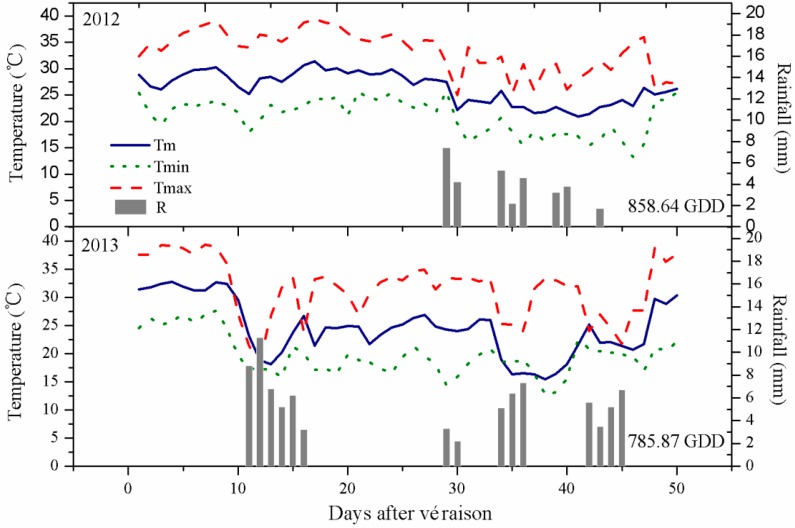
Patterns of mean (T_m_), maximum (T_max_), and minimum (T_min_) daily temperatures and rainfall (R) from véraison to commercial harvest in the study area in 2012 and 2013.

The vineyard in the present study was in a wine-producing region of China with unique ecological conditions, a semi-humid climate, and high rainfall during the berry-ripening stage. The average daily temperature during the first ten days after véraison was higher for the 2013 than the 2012 vintage (Figure 1). The daily maximum temperature exceeded 35 °C for most of this stage, which thus significantly accumulated heat in 2013. 2013 had many more days than 2012 with lower temperatures for 10–50 DAV (days after version). Post-véraison rainfall was higher in 2013 than 2012 (mainly during 10–20 DAV). No rain fell at harvest in 2013, but some rain fell during the final stage of berry ripening (34–45 DAV). Rain began in 2012 at 29 DAV but gradually reduced until 43 DAV. Mathematical formula for calculating the growing degree days (GDD; base 10 °C) was also used [25], and the results showed cumulative growing degree days between véraison and harvest was 858.64 GDD in 2013, lower than 785.87 GDD in 2012. The environmental conditions thus differed between the two vintages.

### 2.2. Effect of the Training Systems on the Vine Microclimates

Vines planted with different training systems are exposed to specific grape-growing conditions [3,4]. The various effects of the vine microclimates, which differ considerably among training systems, require detailed analysis. In our study, thus, temperature, humidity and light intensity in the canopy of SG, VSP and 4AK were measured at three different time points in each year. As shown in Table 1, the light intensity in the canopy of SG was close to the data of VSP, and a little higher temperature and humidity level were showed in VSP than in SG, but no significant difference was shown. Specially, multi-layered canopy of 4AK substantially increased the canopy humidity and temperature, and decreased cluster the solar irradiance.

### 2.3. Effect of the Training Systems on Grape Yield

The initial rationale for studying grape training systems centred primarily on the response of yield to canopy management [5]. In our study, in order to obtain an adequate variability in the grape characteristics for each training system, productive situations were also considered and showed different among three training systems (Table 2). The germination rate and fructification coefficient were higher for VSP and SG than for 4AK, but different training systems had little effect on the percent of bearing branches. The numbers of clusters per vine, average weight per cluster and average yield per vine, however, were 0.55–0.65-fold higher for 4AK than for SG and VSP. These indicators did not differ significantly between SG and VSP but were slightly higher for VSP than SG, similar to previous study for Pinot Noir [26]. Moreover, the training system had a consistent effect on vine yield in the two vintages, consistent with the findings by Reynolds *et al.* [27]. our study suggested that higher numbers of huds and bearing branches (Table S1) contributed to the higher yield of grapevines trained to the 4AK system.

### 2.4. Effect of the Training Systems on the Incidence of Grape Disease

Our experimental field was in a wet area where diseases become serious during fruit ripening due to the high rainfall and humidity. No pesticides were sprayed during the experiment. The diseases were mainly downy mildew on leaves and anthracnose and white rot on berries (Figure S1), and the incidences increased substantially with time.

**Figure 2:**
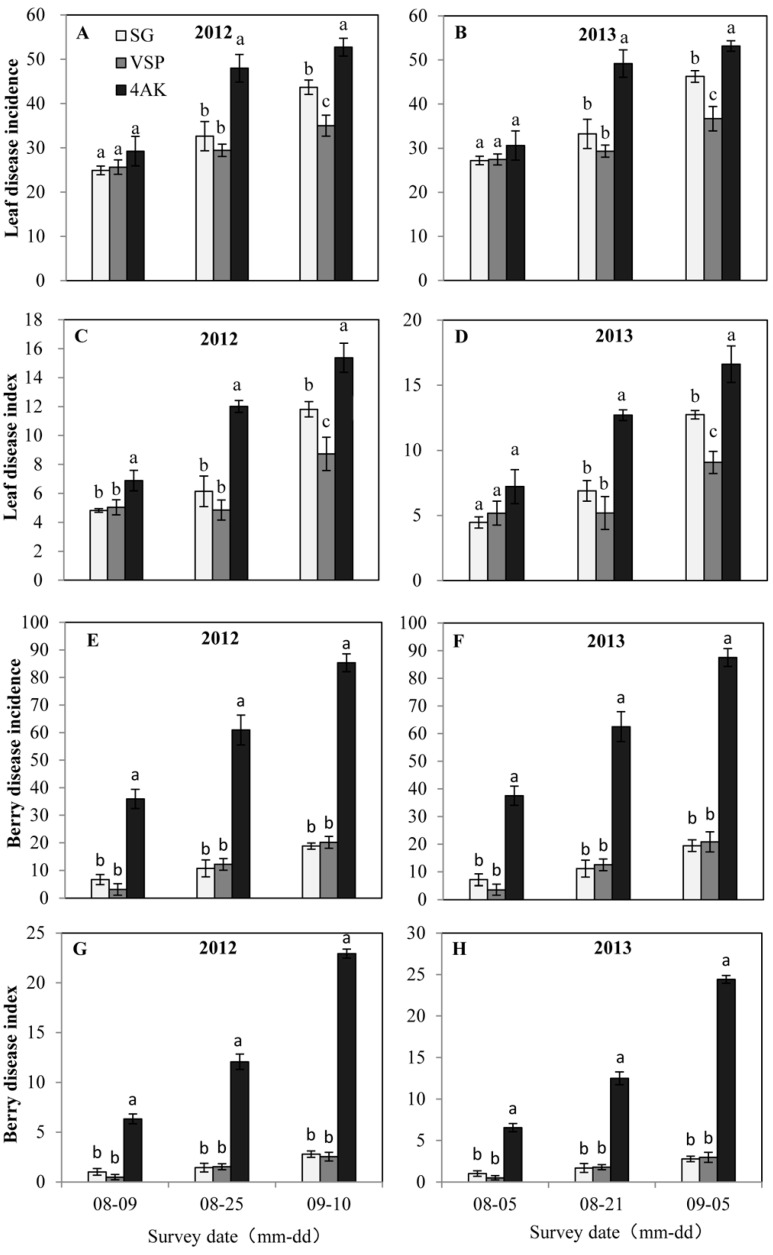
Disease of grape leaves and berries for the three training systems during ripening in 2012 and 2013 vintages. The bars indicate the mean of three replicates and their standard deviations. For the same year, different letters indicate significant differences between treatments calculated by Duncan’s test (*p* < 0.05). (**A**) Leaf disease incidence in 2012; (**B**) Leaf disease incidence in 2013; (**C**) Leaf disease index in 2012; (**D**) Leaf disease index in 2013; (**E**) Berry disease incidence in 2012; (**F**) Berry disease incidence in 2013; (**G**) Berry disease index in 2012; (**H**) Berry disease index in 2013.

Two heavy rains in 2013 led to more serious leaf and berry disease than in 2012 (Figure 2). The incidence of leaf disease and the disease index also differed significantly between SG, VSP, and 4AK during the mid- and final stages but not during the early stage (Figure 2A–D). The incidence of leaf disease and the disease index for 4AK than for SG and VSP were 0.48–0.84-fold and 0.67–1.45-fold higher, respectively, at mid-stage and 0.15–0.30-fold and 0.45–0.83-fold higher, respectively, during the final stage. Referring to the berry diseases, SG and VSP had a lower berry disease incidence and berry disease index of disease throughout the fruit growth (Figure 2E–H), and the disease infection for SG had no significant difference with VSP. Grape disease for 4AK was most serious throughout disease development (Figure 2E–H).

**Table 1:** Microclimatic data of SG, VSP and 4AK vines.

Time			Temperature (°C)			Humidity (%)			Light intensity (×10^5^ lx)		
Vintage	Date (Month-Day)	DAV	SG	VSP	4AK	SG	VSP	4AK	SG	VSP	4AK
2012	8-06 (cloudy)	11	30.1 ± 0.2 b	30.6 ± 1.5 b	32.7 ± 0.9 a	69.5 ± 1.2 b	69.8 ± 1.9 b	74.6 ± 3.4 a	0.152 ± 0.090 a	0.160 ± 0.015 a	0.114 ± 0.043 b
	8-19 (sunny)	24	32.1 ± 0.9 a	31.8 ± 0.4 a	30.6 ± 0.5 b	51.5 ± 0.8 b	51.2 ± 1.5 b	56.5 ± 2.9 a	0.823 ± 0.054 a	0.797 ± 0.093 a	0.526 ± 0.043 b
	9-05 (rainy)	40	25.1 ± 0.4 b	25.9 ± 0.6 b	27.8 ± 0.8 a	88.7 ± 2.9 a	89.1 ± 2.6 a	91.5 ± 3.5 a	0.042 ± 0.007 a	0.047 ± 0.009 a	0.031 ± 0.003 b
2013	8-07 (sunny)	7	35.6 ± 1.1 a	35.9 ± 0.5 a	33.1 ± 1.0 b	59.6 ± 0.3 b	61.1 ± 1.4 b	64.0 ± 0.9 a	1.322 ± 0.073 a	1.414 ± 0.062 a	0.784 ± 0.089 b
	8-15 (rainy)	15	28.6 ± 1.2 b	28.2 ± 0.4 b	32.3 ± 0.7 a	91.2 ± 0.9 b	90.5 ± 1.9 b	94.7 ± 2.1 a	0.034 ± 0.003 a	0.037 ± 0.004 a	0.024 ± 0.008 b
	9-06 (cloudy)	38	24.9 ± 0.4 b	25.3 ± 0.4 b	27.2 ± 0.8 a	70.5 ± 1.9 b	70.6 ± 0.7 b	74.8 ± 2.1 a	0.189 ± 0.052 a	0.168 ± 0.084 b	0.122 ± 0.099 c

Data was measured at 11:00 am to 1:00 pm in each date. Parameters measured for 4AK are an average value of two berry-positioned heights. And results presented are means of two different points in each of three replicates. Different letters within a row for the same year indicate significant differences between treatments calculated by Duncan’s test (*p* < 0.05).

**Table 2:** Effects of the training systems on grape yield in 2012 and 2013.

Factors	2012			2013		
	SG	VSP	4AK	SG	VSP	4AK
Germination rate (%)	85.1 ± 2.8 a	82.7 ± 3.1 a	78.5 ± 2.1 b	80.6 ± 3.9 a	83.4 ± 4.1 a	70.5 ± 3.2 b
Bearing branches/branch (%)	81.4 ± 2.1 a	76.1 ± 1.9 b	81.6 ± 1.5 a	95.0 ± 4.8 a	85.6 ± 2.6 b	87.9 ± 2.3 b
Clusters/vines (n)	17.9 ± 1.7 c	20.3 ± 2.0 b	25.7 ± 2.9 a	18.3 ± 0.9 b	18.9 ± 1.7 b	23.3 ± 2.1 a
Fructification coefficient	1.9 ± 0.2 a	2.0 ± 0.1 a	1.4 ± 0.4 b	1.9 ± 0.2 a	1.5 ± 0.1 b	1.4 ± 0.1 b
Average per cluster weight (g)	123.2 ± 6.3 b	115.1 ± 5.1 b	141.3 ± 8.9 a	108.2 ± 3.7 b	111.7 ± 6.2 b	141 ± 5.2 a
Average yield/vine (kg)	2.2 ± 0.2 b	2.3 ± 0.2 b	3.6 ± 0.4 a	2.0 ± 0.1 b	2.1 ± 0.2 b	3.3 ± 0.5 a

Data represent mean value ±SD for three replicates. Different letters within a row for the same year indicate significant differences between treatments calculated by Duncan’s test (*p* < 0.05).

This is consistent with previous findings [28], likely because of the denser canopy of 4AK with higher temperature and humidity (Table 1). In fact, it is known that high temperature and humidity are the main factors inducing the occurrence and spread of disease within a given vineyard [29,30]. Therefore, although 4AK was the most productive system, it produced a microclimate that is more susceptible for the occurrence and spread of diseases on leaves and berries in a wet region.

### 2.5. Effect of the Training Systems on Berry Maturity at Harvest

Training systems can affect fruit maturity in two ways. Firstly, canopy size, temperature and humidity in the canopy, and the solar irradiance of leaves and fruits directly affect the amount of fruit sugar produced and the titratable acidity [3,31,32]. Secondly, different yielding systems produced fruit with different the soluble solid content [31]. We analysed the amount of reducing sugar, total acids, and the ratio of total soluble sugars to titratable acidity (S/A) of grapes sampled in 2012 and 2013 (Table 3).

**Table 3:** Effect of training system on parameters of berry maturity.

Vintage	Treatment	Sugars (g·L^−1^)	Total Acids (g·L^−1^)	S/A
2012	SG	207.23 ± 3.7 a	5.33 ± 0.21 a	38.98 ± 1.77 a
	VSP	192.13 ± 4.1 b	5.37 ± 0.19 a	35.90 ± 1.04 b
	4AK	199.17 ± 3.8 b	5.70 ± 0.37 a	35.28 ± 1.19 b
2013	SG	202.83 ± 2.8 a	5.04 ± 0.18 a	40.28 ± 0.99 a
	VSP	201.99 ± 2.4 a	5.06 ± 0.22 a	39.92 ± 1.07 a
	4AK	204.03 ± 2.7 a	5.40 ± 0.36 a	37.81 ± 1.52 a

Data represent mean value ± SD for three replicates. Different letters within a column for the same year indicate significant differences between treatments calculated by Duncan’s test (*p* < 0.05). S/A, ratio of total soluble sugars to titratable acidity.

In 2012, the amount of reducing sugar of the juices in SG was significant higher than VSP and 4AK, whichcontributed to the highest S/A, the result agreed with previous study that reducing-sugar content and berry maturity were higher in training systems with lower yields [28,31]. However, in 2013, the sugar content, was higher for 4AK than for SG and VSP, despite the difference was not significant, The difference between the two vintages may have been due to the loss of fruit water caused by the more serious berry disease in 2013 than in 2012 (Figure S2), so that the sugar content in 2013 was higher for 4AK than for SG and VSP. The total-acid content was higher for 4AK than for SG and VSP in both vintages, but the differences between the three training systems were not significant. Generally, different systems, at least in the wet region that our studied, did not altered the berry maturity.

### 2.6. Effect of the Training Systems on the Anthocyanin Composition of the Berry Skins

Grape anthocyanins are natural colorants that are responsible for most of the colour of grapes and young wines [33,34]. Anthocyanins mostly exist in grape skins and the main anthocyanins are five primitive monoglucoside structures and their acetylated or coumaroylated derivatives. Previous studies had reported that anthocyanins are influenced by different training systems [35,36,37].

**Figure 3:**
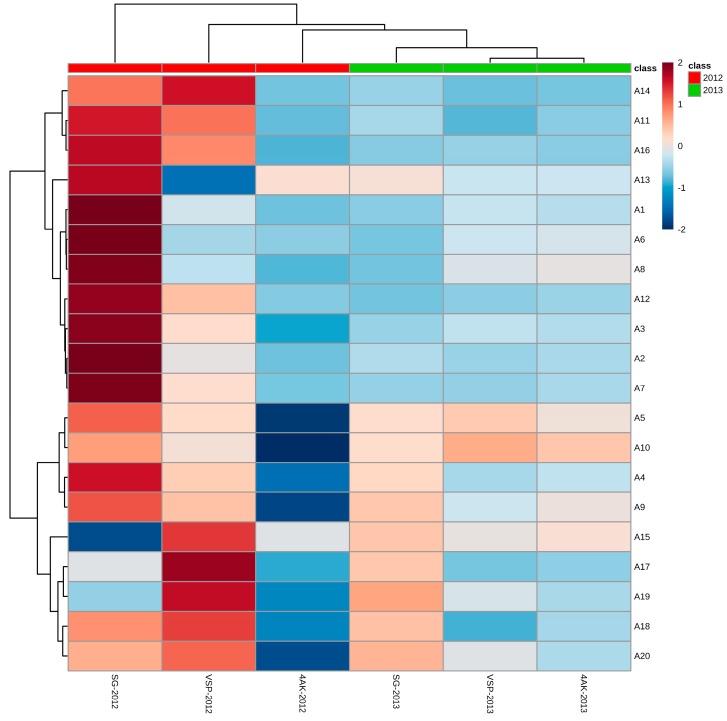
The hierarchically clusteredheat map of individual anthocyanin concentrations with samples in rows and compounds in columns. Color bar defines concentration fold change.

Referring to the difference between three different training systems in our study, as shown in Table 4 and Figure 3, training systems, along with different vintages, strongly affect the amount and profiles of anthocyanins. In particular, the concentrations of total individual anthocyanins, as well as malvidin-3-*O*-glucoside (A5) and Malvidin-3-*O*-(6-*O*-acetyl)-glucoside (A10), which were the most prevalent anthocyanin and the major acylated anthocyanins in harvested berry [38], were higher in SG than VSP and 4AK in 2012, but difference between SG and VSP was not significant. However, in 2013 they showed significantly higher for VSP and showed no significant difference for SG and 4AK. Besides, it was noteworthy that in 2012, despite the major anthocyanins for SG and VSP showed no significant difference, the concentrations of most coumaroylated individual anthocyanins, except delphinidin-3-*O*-(-6-*O*-coumaryl)-glucoside (A11 and A12), cyanidin-3-*O*-(*cis*-6-*O*-coumaryl)-glucoside (A13) and petunidin-3-*O*-(*trans*-6-*O*-coumaryl)-glucoside (A16), were in the order VSP > SG > 4AK in 2012. 4AK produced the lowest concentrations of most individual anthocyanins in both vintages, which was consistent with previous findings that anthocyanin accumulation was affected by cluster yield [39,40].

Besides, light intensity, cluster temperature, and other factors also can have complex influences on the concentration of anthocyanins [41,42,43]. The higher concentrations of most anthocyanins in grape skins from SG and VSP at harvest in each year may associate with the higher amount of sunlight for the thinner canopy of SG and VSP (Table 1).

**Table 4:** Anthocyanin profiles of berry skins for the three training systems in 2012 and 2013 (mg/kg DW).

Anthocyanins	[M^+^]/[M − H]^+^ (Frag. MS^2^ *m*/*z*)	2012			2013		
		SG	VSP	4AK	SG	VSP	4AK
Dephinidin-3-*O*-glucoside(A1)	465 (303)	1011.8 ± 89.45 a	533.49 ± 49.32 b	419.73 ± 33.27 b	446.28 ± 24.38 b	518.82 ± 19.35 a	497.14 ± 21.03 a
Cyanidin-3-*O*- glucoside(A2)	449 (287)	268.32 ± 24.67 a	132.83 ± 11.29 b	88.62 ± 9.48 c	110.47 ± 9.77 a	101.87 ± 7.14 a	107.82 ± 8.31 a
Petunidin-3-*O*- glucoside(A3)	479 (317)	1551.66 ± 160.32 a	1060.2 ± 100.42 b	731.31 ± 51.09 c	864.86 ± 49.12 a	930.16 ± 34.33 a	904.14 ± 32.91 a
Peonidin-3-*O*- glucoside(A4)	463 (301)	1050.28 ± 83.22 a	857.19 ± 79.87 b	576.88 ± 43.44 c	843.29 ± 46.41 a	739.98 ± 35.79 b	762.12 ± 31.11 b
Malvidin-3-*O*-glucoside(A5)	493 (331)	6563.04 ± 532.3 a	5703.17 ± 233.89 a	3719.25 ± 244.5 b	5659.64 ± 99.3 b	5863 ± 100.2 a	5554.96 ± 93.3 b
Delphinidin-3-*O*-(6-*O*-acetyl)-glucoside(A6)	493 (331)	325.23 ± 22.1 a	142.66 ± 10.36 b	133.56 ± 10.22 b	125.28 ± 8.77 b	158.88 ± 9.38 a	164.41 ± 11.31 a
Cyanidin-3-*O*-(6-*O*-acetyl)-glucoside(A7)	491 (287)	94.45 ± 8.22 a	51.28 ± 4.11 b	31.73 ± 2.79 c	35.43 ± 2.54 a	34.9 ± 3.01 a	37.49 ± 2.90 a
Petunidin-3-*O*-(6-*O*-acetyl)-glucoside(A8)	521 (317)	985.88 ± 79.49 a	548.2 ± 49.32 b	449.4 ± 33.21 b	473.14 ± 34.77 b	582.44 ± 57.90 a	597.41 ± 58.33 a
Peonidin-3-*O*-(6-*O*-acetyl)-glucoside(A9)	505 (301)	640.24 ± 45.77 a	572.74 ± 42.89 a	375.78 ± 30.21 b	568.99 ± 28.86 a	517.69 ± 19.77 b	535.19 ± 17.73 b
Malvidin-3-*O*-(6-*O*-acetyl)-glucoside(A10)	535 (331)	7174.56 ± 567.44 a	6384.86 ± 484.44 a	3865.15 ± 330.23 b	6507.66 ± 122.01 b	7058.84 ± 114.56 a	6790.81 ± 79.30 b
Delphinidin-3-*O*-(*cis*-6-*O*-coumaryl)-glucoside(A11)	611 (303)	4.02 ± 0.32 a	3.58 ± 0.21 a	2.17 ± 0.19 b	2.44 ± 0.10 a	2.14 ± 0.11 b	2.32 ± 0.09 a
Delphinidin-3-*O*-(*trans*-6-*O*-coumaryl)-glucoside(A12)	611 (303)	63.18 ± 5.77 a	47.68 ± 3.92 b	36.12 ± 2.87 b	35.14 ± 3.21 a	36.55 ± 2.28 a	37.48 ± 3.10 a
Cyanidin-3-*O*-(*cis*-6-*O*-coumaryl)-glucoside(A13)	595 (287)	2.36 ± 0.19 a		1.52 ± 0.11 b	1.5 ± 0.10 a	1.33 ± 0.07 b	1.34 ± 0.05 b
Cyanidin-3-*O*-(*trans*-6-*O*-coumaryl)-glucoside(A14)	595 (287)	31.72 ± 3.09 a	36.4 ± 3.21 a	18.01 ± 1.78 b	19.6 ± 0.82 a	17.77 ± 0.60 b	18.13 ± 0.52 b
Petunidin-3-*O*-(*cis*-6-*O*-coumaryl)-glucoside(A15)	625 (317)		15.79 ± 1.02 a	8.82 ± 0.52 b	11.2 ± 0.77 a	9.12 ± 0.34 b	9.7 ± 0.52 b
Petunidin-3-*O*-(*trans*-6-*O*-coumaryl)-glucoside(A16)	625 (317)	222.22 ± 17.34 a	193.94 ± 16.21 a	133.6 ± 9.88 b	141.81 ± 8.35 a	144.83 ± 7.19 a	142.75 ± 4.02 a
Peonidin-3-*O*-(*cis*-6-*O*-coumaryl)-glucoside(A17)	609 (301)	23.52 ± 2.01 b	31.46 ± 2.34 a	20.07 ± 1.90 b	25.44 ± 1.92 a	21.08 ± 2.02 b	21.64 ± 1.27 b
Peonidin-3-*O*-(*trans*-6-*O*-coumaryl)-glucoside(A18)	609 (301)	290.73 ± 20.11 a	309.58 ± 23.41 a	211.76 ± 18.78 b	276.59 ± 18.34 a	227.41 ± 14.30 b	243.18 ± 10.20 b
Malvidin-3-*O*-(*cis*-6-*O*-coumaryl)-glucoside(A19)	639 (331)	137.15 ± 14.22 b	236.79 ± 24.90 a	104.89 ± 9.11 c	192.65 ± 16.30 a	155.37 ± 12.39 b	143.23 ± 12.90 b
Malvidin-3-*O*-(*trans*-6-*O*-coumaryl)-glucoside(A20)	639 (331)	1916.42 ± 156.66 a	2055.69 ± 178.44 a	1245.98 ± 104.89 b	1900.48 ± 97.23 a	1723.18 ± 76.34 b	1639.2 ± 55.45 b
Total concentration		22,380.3 ± 2017.82 a	18,901.74 ± 1591.05 a	12,165.53 ± 979.72 b	18,230.69 ± 244.34 b	18,778.45 ± 267.73 a	18,200.76 ± 200.41 b

Data represent mean value ±SD for three replicates. Different letters within a row for the same year indicate significant differences between treatments calculated by Duncan’s test (*p* < 0.05). [M^+^], molecular ion; [M − H] ^+^, fragment ion; DW, dry weight.

### 2.7. Effect of Three Training Systems on the Modified Anthocyanins

**Figure 4:**
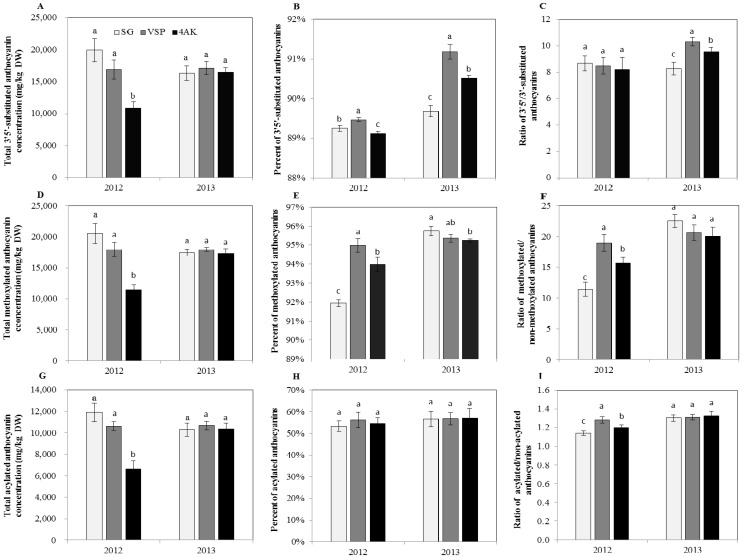
The concentrations and ratios of various anthocyanins in grape berry skins for the three training systems in 2012 and 2013. (**A**) The concentration of 3′5′-substituted anthocyanins; (**B**) The percent of 3′5′-substituted anthocyanins; (**C**) The ratio of 3′5′/3′ -substituted anthocyanins; (**D**) The concentration of methoxylated anthocyanins; (**E**) The percent of methoxylated anthocyanins; (**F**) The ratio of methoxylated/non-methoxylated anthocyanins; (**G**) The concentration of acylated anthocyanins; (**H**) The percent of acylated anthocyanins; (**I**) The ratio of acylated/non-acylated anthocyanins. DW, dry weight.

F3′H (flavonoid 3′-hydroxylase) and F3′5′H (flavonoid 3′5′-hydroxylase) are involved in the biosynthetic pathway of cyanidin- and delphinidin-based anthocyanins, respectively. The cyaniding-based anthocyanins, which are called 3′-substituted anthocyanins, include cyaniding and peonidin monoglycosides and their acylated derivatives, and the delphinidin-based anthocyanins, which are called 3′5′-substituted anthocyanins, are made up of glycosylated forms of delpinidin, petunidin, malvidin and their acylated derivates [44]. A higher accumulation of 3′5′-substituted anthocyanins in grape skins is expected to produce more intensely purple berries and darker red wines [45,46]. In our study, 3′5′-substituted anthocyanins accounted for 89.12%–91.18% of all anthocyanins (Figure 4B). Among three treatments, the results showed that SG and VSP had the highest concentrations of total 3′5′-substituted anthocyanins in 2012 and 2013, respectively (Figure 4A). The percent of 3′5′-substituted anthocyanins in VSP was significantly higher than SG and 4AK in two vintages, and the ratio of 3′5′/3′-substituted anthocyanins in VSP also showed significantly higher levels in 2013 (Figure 4C). VSP thus produced darker berries than SG and 4AK.

Methoxylation on the position 3′and 5′ of the anthocyanin B-ring has a slight reddening effect on the color of anthocyanins [47,48]. *O*-methoxylation of the 3′ position of cyanidin and delphinidin leads to the formation of peonidin and petunidin, and *O*-methoxylation of the 3′ and 5′ positions of delphinidin leads to the formation of malvidin. The methylated anthocyanins, including peonidin, petunidin, malvidin anthocyanins and their derivatives, are relatively stable and represent the major pool of anthocyanins in mature berries [49]. Besides, anthocyanin acyltransferases (AATs) can catalyze the corresponding acyl transfer from acyl-CoA to the glycosyl moiety of anthocyanins. In the skins of Cabernet Sauvignon berries, acetylation and cinnamylation of anthocyanins caused the production of two types of acylated anthocyanins [38]. And among them, the modification of acylation makes anthocyanins more stable and bluer or enhances the pigment solubility in water [50]. In our study, methoxylated and acylated anthocyanins accounted for 91.94%–95.75% and 53.28%–57.02%, respectively (Figure 4E,H). Just like 3′5′-substituted anthocyanins, the concentrations of methoxylated and acylated anthocyanins were also in the order SG > VSP > 4AK in 2012 and VSP > SG > 4AK in 2013 (Figure 4D,G). With regard to the ratios of the methoxylated/non-methoxylated and acylated/non-acylated anthocyanins, as well as the percent of methoxlated anthocyanins, they were significantly higher for VSP than for SG and 4AK in 2012, but the difference between three training systems was not significant in 2013 (Figure 4E,F,I).

These results indicated that among three training systems, VSP was more conducive to accumulate methoxylated and acylated anthocyanins in a relative dry vintage. The different modification of the individual anthocyanins among three training systems should be contributed to previous report that the modification of anthocyanin profiles of the grapes depended on yields and environmental conditions [51]. Nevertheless, considering the similar microclimate shared by the berries from SG and VSP, the significant differences of the anthocyanin profiles for the two systems may be contributed to different yield, after all, the proportions of individual anthocyanins was indeed modified by the variation of yield [39,52].

### 2.8. PLS-DA Analysis of the Concentrations of Individual Anthocyanin in Two Vintages

**Figure 5:**
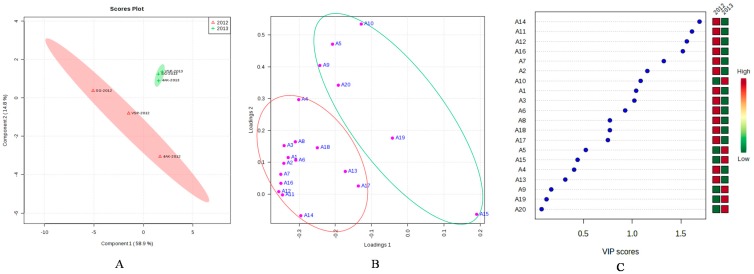
Results of discrimination of the grape samples from three training systems by the concentrations of individual anthocyanin detected in two vintages using PLS-DA analysis. (**A**) Scores plot; (**B**) Loading plot; (**C**) Selected compounds base on VIP scores.

Seasonal vintage variation can influence anthocyanin accumulation [41], whereas others reported there was minimal influence of the season [53]. Besides, individual anthocyanin concentration varied as a consequence of chemical structure, canopy management practices, and seasonal climatic conditions [51]. Thus, in this study PLS-DA analysis of the characteristic components of grape samples was conducted based on the concentrations of individual anthocyanin from three training systems detected in two vintages (Figure 5). Twenty different anthocyanins detected at harvest in two vintages were used as the variables. Leave one out cross-validation (LOOCV) method was used for cross-validation. As seen from Figure 5A, three grapevine training systems in 2012 were clearly separated from that in 2013. Also, separating efficiency of the vintages of 2012 on different training systems was more obvious than that of 2013. Correspondingly, the characteristic components of each vintage were found in the loading plots (Figure 5B), and the selected compounds base on VIP scores were shown in Figure 5C. Because of their phenolic B ring substitution, peonidin, malvidin and their acylated derivatives are relatively stable and represent the major anthocyanin pools in mature grapes [50]. Compared with 2012, vintage of 2013 was more advantages to produce stable anthocyanins, including malvidin-3-*O*-(6-*O*-acetyl)-glucoside (A10), malvidin-3-*O*-glucoside (A5), and malvidin-3-*O*-(*cis/trans*-6-*O*-coumaryl)-glucoside (A19, A20), but decreased the concentrations of cyanidin-3-*O*-(*trans*-6-*O*-coumaryl)-glucoside (A14), Delphinidin-3-*O*-(*cis*-6-*O*-coumaryl)-glucoside (A11), delphinidin-3-*O*-(*trans*-6-*O*-coumaryl)-glucoside (A12), Petunidin-3-*O*-(*trans*-6-*O*-coumaryl)-glucoside (A16), cyanidin-3-*O*-(6-*O*-acetyl)-glucoside (A7), *etc.* Generally, low temperatures, such as 25 °C, favor the anthocyanin biosynthesis [12], Whereas, high temperatures, such as 35 °C are associated with anthocyanin degradation and inhibition of anthocyanin accumulation [12], the moderated temperature appeared to lead to higher concentrations of delphindin, cyaniding, petunidin and peonidin [43]. Considering the climatic parameters, including mean temperature, the maximum, and minimum temperature, the moderate temperature of 25 °C was more frequent in 2012, also, the extreme temperature (>35 °C) were more frequent in 2013 than in 2012 during verasion (Figure 1), so the increase of cyaniding, dephinidin, petunidin and their acylated derivatives in 2012 could be attributed to the favourable temperature. Besides, a wet vintage of 2013 with a higher temperature at verasion was more advantageous to accumulate higher proportion of 3′5′-substituted and acylated anthocyanins, which was consistent with previous report that higher temperature led to a higher concentration and a higher proportion of total skin anthocyanins comprised by malvidin-based anthocyanins, driven primarily by increases in the acylated derivatives [43]. That means the content of non-acylated anthocyanins was influenced most by the higher temperature, so that in our study quite higher percent of anthocyanins reported 3′5′-substituted was showed, also, malvidin-3-*O*-(6-*O*-acetyl)-glucoside had higher levels than malvidin-3-*O*-glucoside (Table 4), which were inconsistent with previous results [54]. Above anthocyanin analysis (Table 4 and Figure 3) suggested that anthocyanin compositions were also related to the different weather conditions of the growing season.

## 3. Experimental Section

### 3.1. Experimental Field Site

A field experiment was conducted in a commercial vineyard in Jingyang, Shaanxi, China (34°65′N, 108°75′E) during the 2012 and 2013 growing seasons. This region belongs to continental monsoonal climate with 2195.2 h of sunshine annually and an average frost-free period of 213 days. The mean annual temperature and precipitation are 13 °C and 548.7 mm, respectively, and the main precipitation and high temperature were mainly concentrated in the grapes of growing season. The vineyard soil is classified as sandy loam.

### 3.2. Experimental Design and Berry Sampling

Grapevines (cv. Cabernet Sauvignon) vines were planted in 2009 and grown on their own roots in a north-south orientation. The technology of drip-irrigation was used to easily control the amount of irrigation, especially when the water demand was feeble. The experimental design was a randomised complete block for three replications with 10-vine plots for each, and at the time of planting, the vines were separately trained to form three different training systems: Single Guyot (SG), which has one cane at 50 cm above ground and all shoots are positioned into catch wires on vertical trellis (Figure S3); Spur-pruned Vertical Shoot-Positioned (VSP), the bilateral cordon system with two strong horizontal cordons at 50 cm above ground and spur-pruned with vertically trained shoots (Figure S4); and Four-Arm Kniffin (4AK), vertical trunk with the upper pair of arms at 60 cm and the under pair of arms at 40 cm above ground (Figure S5). Parameters of SG, VSP and 4AK vines, shoots and clusters were examined on five trees (Table S1). All viticultural practices followed standard commercial vineyard practices were identical for all experimental vines. The increase in concentration of total soluble soild is a reliable indicator for the progress of berry ripening [15], thus, using a using a PAL-1 digital refractometer (Atago, Tokyo, Japan) to monitor the accumulation of solule sugar were used to assess berry maturity and decide the harvest date. At fruit maturity (Total soluble solids (°Brix) > 22%, 14 September 2012, 19 September 2013), three hundred berries were randomly sampled from each treatment. One hundred of the berries were crushed into juice for determining the amount of reducing sugar and the titratable acidity, and the other 200 berries were frozen and stored at −80 °C until the analysis of the anthocyanin compounds.

### 3.3. Meteorological Survey

The meteorological data of the region for 2012 and 2013 were obtained from the local meteorological administration, and temperature and rainfall were recorded daily. Measurements of the microclimatic data of SG, VSP and 4AK vines were made at the mid-day of 6 August 2012 (sunny day), 26 August 2012 (cloudy day), 5 September 2012 (rain day), 7 August 2013 (sunny day), 15 August 2013 (rainy day), and 6 September 2013 (cloudy day), respectively. Humidity and temperature in each canopy were determinated using a commercially available Kestrel 4500 meteorological package (Pocket Weather Tracker, Nielsen-Kellerman, Boothwyn, PA, USA), at each chosen date, this device was placed inside the vine foliage in the cluster’s zone of each training system. Light intensity was measured with a ZDS-10-type automatic shift digital luxmeter (Shanghai Jiading Xuelian Meter Factory, Shanghai, China), and it was also placed at the position of randomly chosen clusters. Each biological replicate of each training system was subjected to two independent measurements.

### 3.4. Grape Yield

Investigations of grape yield factors were conducted in 10 vines of each replicate. In particular, the numbers of buds, branches, vegetative branches, and bearing branches were recorded after germination and inflorescence separation, germination rate and percentage of bearing branches were then calculated. At berry technical maturity, the number of clusters per vine was recorded and all clusters in each treatment were weighed, then fructification coefficient, the average cluster weight, and average yield per vine were calculated by following formulas:

(1) $$\text{Fructification coefficient }\left(\text{\%}\right)\text{ = }\frac{\text{Number of clusters per vine}}{\text{Number of bearing branches per vine}}\text{ × 100}$$

(2) $$\text{Average weight per cluster }\left(\text{g}\right)\text{ = }\frac{\text{Total weight of all clusters in each treatment}}{\text{Total number of all clusters in each treatment}}$$

(3) Average yield per vine =Average weight per cluster ×number of cluster per vine

### 3.5. Incidence of Grape Disease

Diseases were identified by visual inspection and monitored during their early (9 August 2012, 5 August 2013), peak (25 August 2012, 21 August 2013), and final (10 September 2012, 05 September 2013) stages. Thirty random shoots and clusters from different parts of vines were examined for the incidence of leaf and berry disease, respectively, and for disease indexing [2]. The extents of leaf and berry disease were graded with the Desaymard 0–10 (Table 5) and the 0–7 (Table 6) classification scales, respectively [55,56]. Disease incidence and the disease index were calculated by:

(4) $$\text{Disease incidence }\left(\text{\%}\right)\text{ = }\frac{\text{Number of infected leaves (clusters)}}{\text{Total number of leaves (clusters)}}\text{ × 100\%}$$

(5) $$\text{Disease index = }\frac{\sum^{\text{​}}\left[\text{Diseases grade ×number of infected leaves (clusters)}\right]}{\text{Total number of leaves }\left(\text{clusters}\right)\text{ ×highest disease grade}}\text{ × 100}$$

**Table 5:** Desaymard 0–10 classification scale for leaf disease.

Grade	Leaf Disease Spot Area (%)
0	0
1	0.1–2.5
2	2.6–5.0
3	5.1–15.0
4	15.1–30.0
5	30.1–50.0
6	50.1–70.0
7	70.1–85.0
8	85.1–95.0
9	95.1–97.5
10	97.6–100

**Table 6:** 0–7 classification scale for berry disease.

Grade	Infected Berries per Cluster (%)
0	0
1	<5
2	5.1–15.0
3	15.1–30.0
4	30.1–45.0
5	45.1–65.0
6	65.1–85.0
7	>85

### 3.6. Reducing Sugar and Titratable Acidity

The amount of reducing sugar and the titratable acidity of the juice were determined by Fehling reagent titration and sodium hydroxide titration, respectively, following the national standards of the People’s Republic of China [57]. Specially, titratable acidity was measured by titration to pH 8.2 and expressed as tartaric acid equivalent.

### 3.7. Anthocyanin Compositions

The anthocyanins in the grape skins were extracted following the methods of He *et al.* [44]. Freeze-dried skins (0.50 g, dry weight (DW)) were ground in triplicate, weighed into 50 mL centrifuge tubes with 10 mL of solvent (methanol/acetic acid, 98:2, *v*/*v*), ultrasonicated for 10 min, and then shaken on an orbital shaker (SHZ-88A, Taicang Experiment Equipment Factory, Jiangsu, China) at 130 rpm for 30 min at 25 °C. The supernatant was centrifuged at 1800× *g* for 5 min at 4 °C and decanted into 50 mL centrifuge tubes, and then the precipitate was re-extracted three times with the same solvent (10 mL). The supernatants were combined and evaporated to dryness in a rotary evaporator (SENCO-R series; Shanghai Shensheng Biotech Co. Ltd., Shanghai, China) at 35 °C under vacuum. The dried residuals were re-dissolved in 10 mL of sample buffer consisting of a 9:1 (*v*/*v*) A:B mobile phase. Phase A was 6% (*v*/*v*) acetonitrile containing 2% (*v*/*v*) formic acid, and phase B was 54% (*v*/*v*) acetonitrile containing 2% (*v*/*v*) formic acid. The solution was filtered through a 0.45 μm organic membrane and then used for qualitative and quantitative analyses by high-performance liquid chromatography (HPLC) with diode array detection and electrospray ionization/mass spectrometry (DAD/ESI-MS).

### 3.8. HPLC-DAD/ESI-MS Analysis of Anthocyanin Compounds

The anthocyanins were chromatographically analysed using an Agilent 1100 series LC-MSD trap VL (Agilent, Santa Clara, CA, USA) equipped with a G1379A degasser, G1312BA Quatpump, G1313A ALS autosampler, G1316A column, G1315A DAD, and reversed-phase column (Kromasil C18, 250 × 4.6 mm id, 5 μm particle size, Restek corporation, Bellefonte, PA, USA). The mobile phase A was 6% (*v*/*v*) acetonitrile and 2% (*v*/*v*) formic acid in water, and B was 54% (*v*/*v*) acetonitrile containing 2% (*v*/*v*) formic acid in water. The proportions of solvent B varied as follows: 1–18 min, 10%–25%; 18–20 min, 25%; 20–30 min, 25%–40%; 30–35 min, 40%–70%; and 35–40 min, 70%–100%. The column was held at 50 °C and was flushed at a flow rate of 1.0 mL·min^−1^. The injection volume was 30 μL, and the detection wavelength was 525 nm. MS conditions were: electrospray ionisation (ESI) interface, positive ion model, 30 psi nebuliser pressure, 12 mL·min^−1^ dry gas flow rate, 300 °C dry gas temperature, and scans between *m*/*z* 100 and 1500 [38]. All anthocyanin compounds were identified by comparing their order of elution and retention time with those of standards, and the weight of molecular ion and the fragment ion were compared with standards and references [2,58]. Quantitative determinations used the external-standard method with commercial standards. The calibration curves were obtained by injection of standard solutions under the same conditions as for the samples over the range of concentrations observed. The compounds for which no standards were available were quantified with the curves of various nonacylated anthocyanins. Each acylated anthocyanin was thus expressed as relative dephinidin-3-*O*-glucoside, cyanidin-3-*O*-glucoside, petunidin-3-*O*-glucoside peonidin-3-*O*-glucoside, and malvidin-3-*O*-glucoside equivalence per kilogramme dry weight. All analyses were performed in triplicate.

### 3.9. Chemicals and Standards

The standards, dephinidin-3-*O*-glucoside, cyanidin-3-*O*-glucoside, petunidin-3-*O*-glucoside, peonidin-3-*O*-glucoside, and malvidin-3-*O*-glucoside, were purchased from Sigma-Aldrich Co. (St. Louis, MO, USA). HPLC-grade methanol, formic acid, and acetonitrile were purchased from Fisher (Fairlawn, NJ, USA). Analytical-grade methanol and formic acid were purchased from the Beijing Chemical Reagent Plant (Beijing, China).

### 3.10. Statistical Analysis

Data were analysed by Microsoft Excel 2007 and were represented as means of the triplicate experiments. One-way analysis of variance (ANOVA) and Duncan’s multiple-range tests used SAS 9.2 software (SAS Institute Inc., Cary, NC, USA) to determine the significance of differences among samples, at a significance level of 0.05. Principal component analysis (PCA) and Partial least square discriminant analysis (PLS-DA) were performed by Metabo-Analyst 3.0. Auto-scaling was used in normalization procedure [59].

## 4. Conclusions

We investigated grape yield, the incidence of grape diseases, and the anthocyanin profiles in the berry skins of *Vitis vinifera* cv. Cabernet Sauvignon grapes for three training systems: Single Guyot (SG), Vertical Shoot-Positioned (VSP), and Four-Arm Kniffin (4AK). In comparison to SG and VSP, 4AK was the most productive system, but it had the more serious incidence of diseaseson leaves and berries. Seasonal vintage influenced the effect of training systems on anthocyanin accumulation, the relatively dry vintage could well discriminate three training systems, but a wet vintage was more advantageous to accumulate 3′5′-substituted and acylated anthocyanins.Between three training systems, 4AK produced the lowest concentration of total individual anthocyanins in both vintages. SG just provided better grape maturity and higher levels of concentrations of total individual anthocyanins than VSP in 2012, but not in a wetter vintage of 2013. in 2013, grapes from VSP system had significantly highest concentrations of total individual anthocyanins, malvidin-3-*O*-glucoside and malvidin-3-*O*-(6-*O*-acetyl)-glucoside. With regard to the anthocyanin stability, the significantly higher levels of the proportations of 3′5′-substituted, methoxylated anthocyanins, as well as the ratios of 3′5′/3′-substituted, methoxylated/non-methoxylated and acylated/non-acylated anthocyanins, were also showed in VSP system. In summary, the VSP system allowed the cultivation of grapes in a wet region with a low incidence of disease and superior berry quality with more stable anthocyanins.

## Supplementary Materials

Supplementary materials can be accessed at: http://www.mdpi.com/1420-3049/20/10/18967/s1.
