# Supplementary material for: Effect of Three Training Systems on Grapes in a Wet Region of China: Yield, Incidence of Disease and Anthocyanin Compositions of Vitis vinifera cv. Cabernet Sauvignon
Source: Molecules. 2015 Oct 19;20(10):18967–87. doi: 10.3390/molecules201018967 (PMC6332443; doi:10.3390/molecules201018967)
Supplement: Supplementary file 1 [file molecules-20-18967-s001.pdf]

Supplementary Material

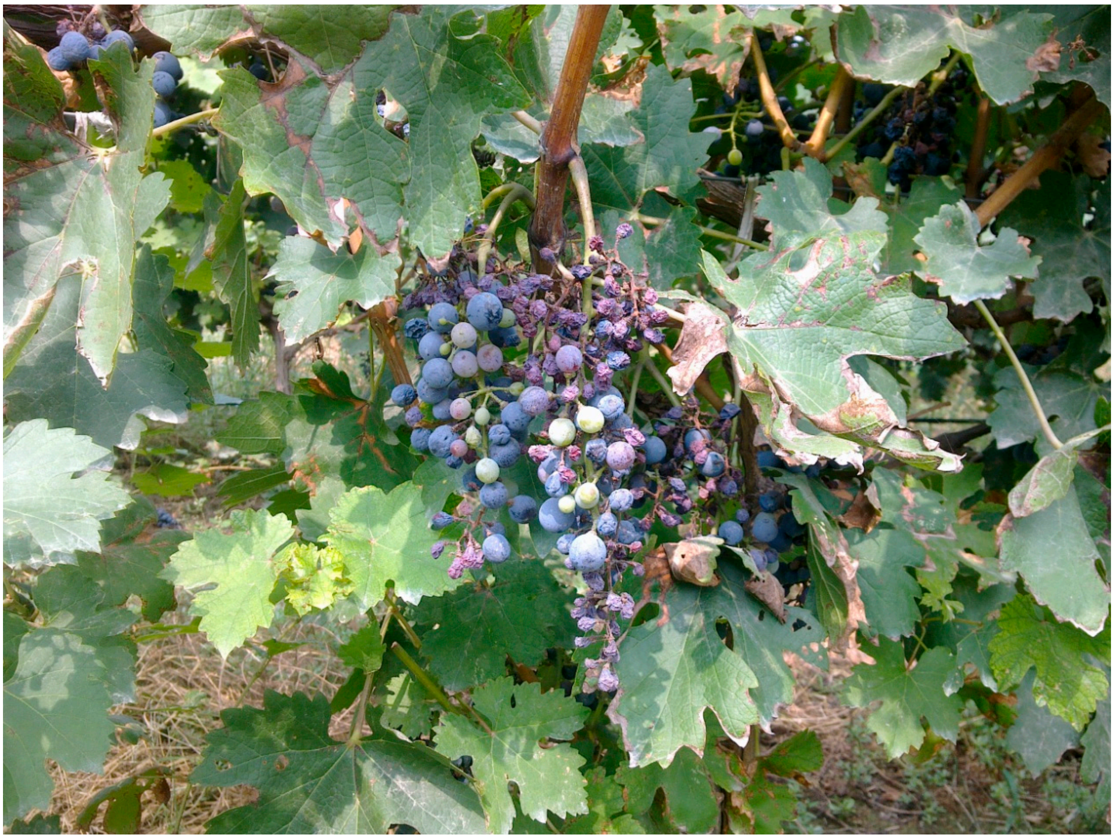

Figure S1. The picture of grape disease on leaves and berries.

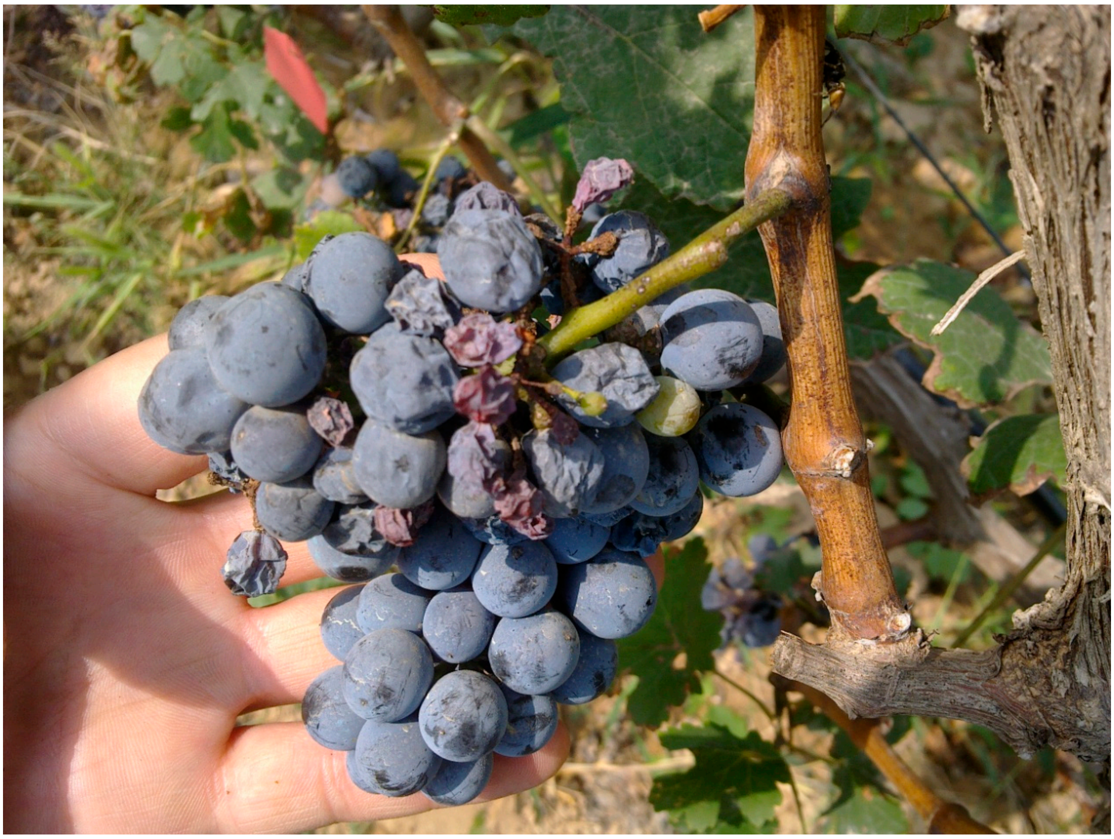

Figure S2. The picture of berry shrinkage caused by serious disease in 2013.

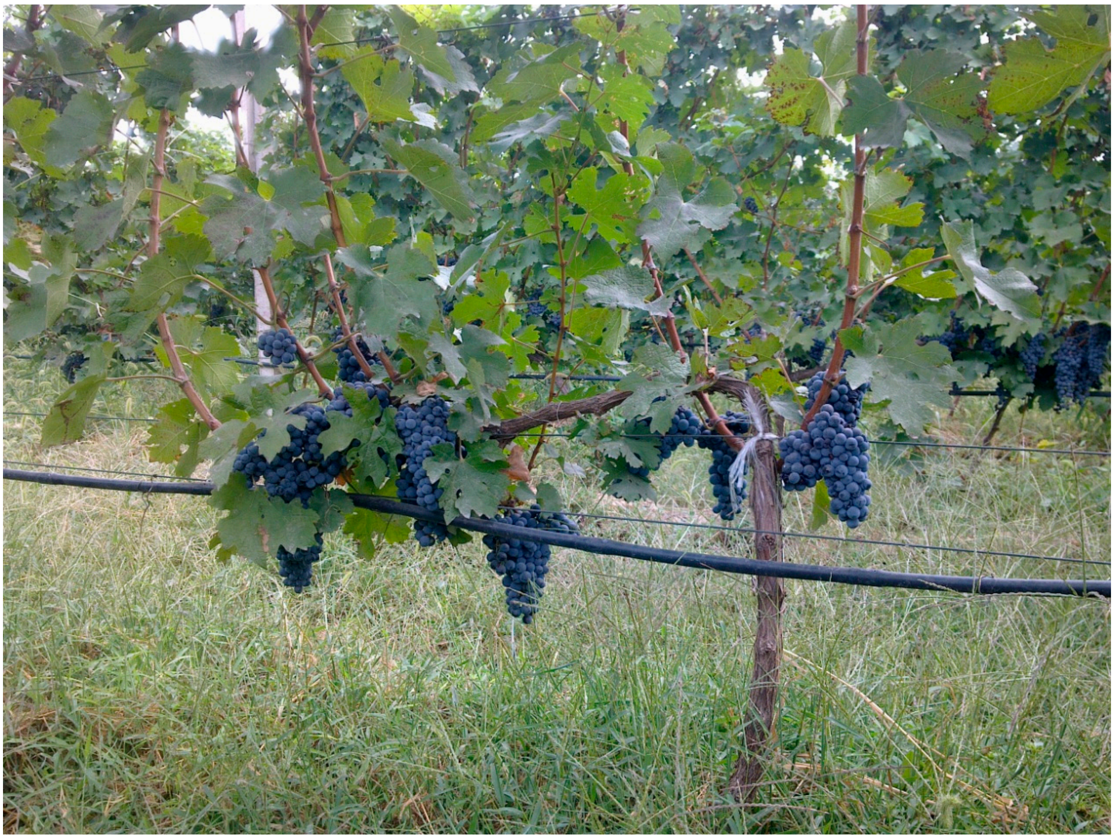

Figure S3. The picture of Single Guyot (SG) training system.

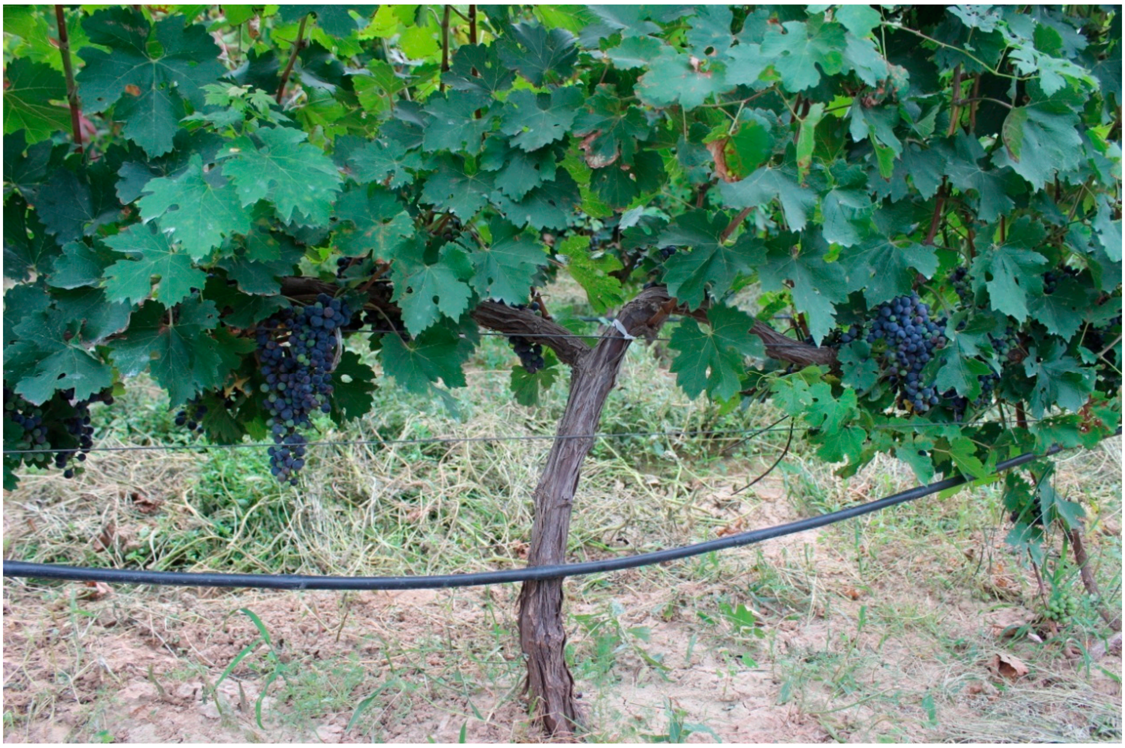

Figure S4. The picture of Spur-pruned Vertical Shoot-Positioned (VSP) training system.

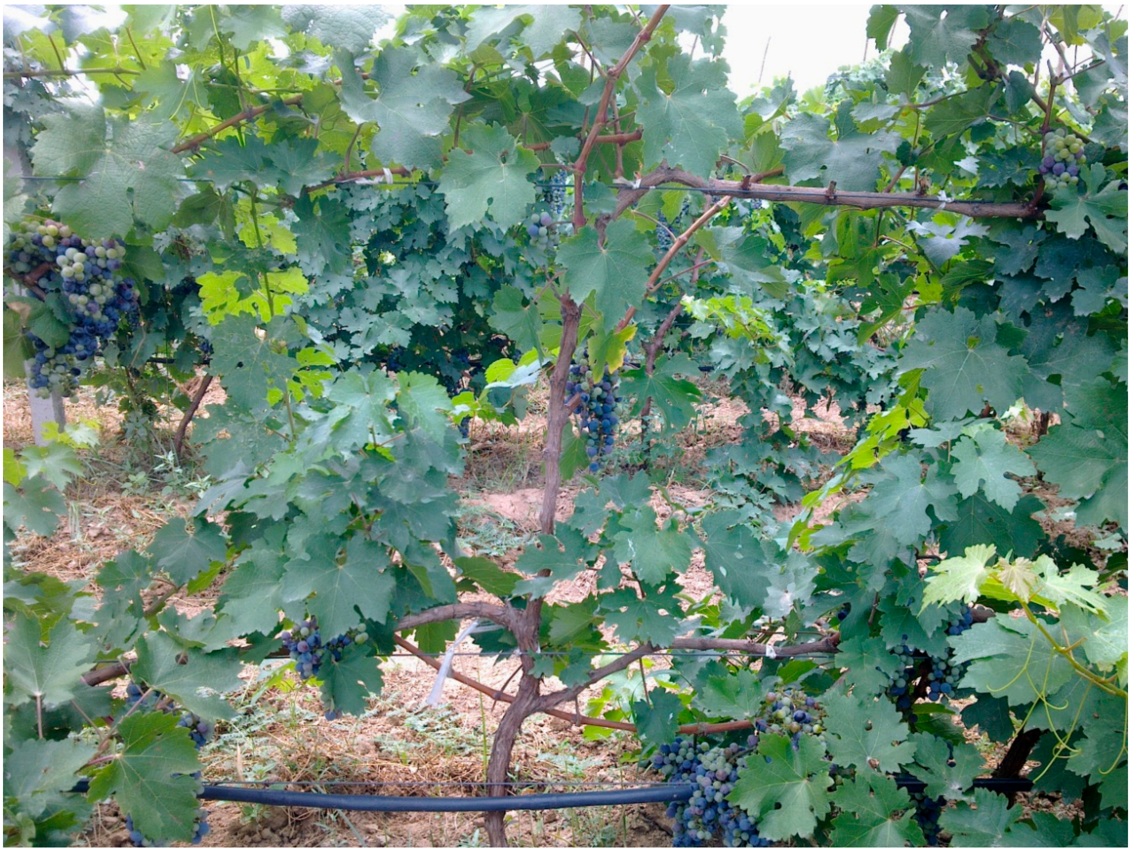

Figure S5. The picture of Four-Arm Kniffin (4AK) training system.

Table S1. Parameters of SG, VSP and 4AK vines, shoots and clusters.

Parameter	Vintage	SG	VSP	4AK
Canopy height (m)	2012/2013	1.3	1.3	1.5
Canopy width (m)	2012/2013	0.7	0.7	0.7
Distance between rows (m)	2012/2013	2.0	2.0	2.0
Buds/vine (n)	2012	13.3 ± 1.4 b	16.2 ± 1.9 b	28.4 ± 2.6 a
	2013	12.4 ± 2.4 c	17.6 ± 2.9 b	27.1 ± 4.1 a
Branches/vine (n)	2012	11.3 ± 0.9 b	13.4 ± 1.3 b	22.3 ± 2.7 a
	2013	10.0 ± 0.7 c	14.6 ± 2.3 b	19.1 ± 2.8 a
Vegetative branches/vine (n)	2012	2.1 ± 0.3 c	3.2 ± 0.3 b	4.1 ± 0.4 a
	2013	1.4 ± 0.2 b	2.3 ± 0.3 a	2.3 ± 0.5 a
Bearing branches/vine (n)	2012	9.2 ± 1.1 b	10.2 ± 1.1 b	18.2 ± 2.3 a
	2013	9.5 ± 1.3 c	12.5 ± 0.9 b	16.8 ± 2.1 a
Clusters/ bearing branch (n)	2012	1.6 ± 0.2 a	1.5 ± 0.1 a	1.2 ± 0.1 b
	2013	1.8 ± 0.3 a	1.3 ± 0.1 b	1.2 ± 0.1 b
Clusters/vines (n)	2012	17.9 ± 1.7 c	20.3 ± 2.0 b	25.7 ± 2.9 a
	2013	18.3 ± 0.9 b	18.9 ± 1.7 b	23.3 ± 2.1 a

Results presented are Means ± SD of five vines in each of three replicates. Different letters within a row indicate significant differences between treatments calculated by Duncan's test ($p < 0.05$).
